# Simplified and Refined Murine Model of Reversible Aortic Constriction for Characterizing Cardiac Functional Recovery

**DOI:** 10.1016/j.jacbts.2026.101575

**Published:** 2026-06-22

**Authors:** Elnaz Ghajar-Rahimi, Pierre Sicard, Ava Flynn, Laura Castel, Craig J. Goergen, Thomas Moore-Morris

**Affiliations:** a Weldon School of Biomedical Engineering, Purdue University, West Lafayette, Indiana, USA; b Univ Montpellier, INSERM, CNRS, PhyMedExp, Montpellier, France; c IGF, Univ Montpellier, CNRS, Inserm, Montpellier, France

Murine models of transverse aortic constriction (TAC) followed by debanding (deTAC) are commonly used to investigate mechanisms driving functional recovery in a controlled setting.^1^ However, TAC-deTAC currently requires 2 consecutive thoracotomies, increasing surgical risk and variability.^2^ To address these limitations, we developed a reproducible modification of the TAC procedure that enables timed and minimally invasive aortic debanding.

**Figure 1:**
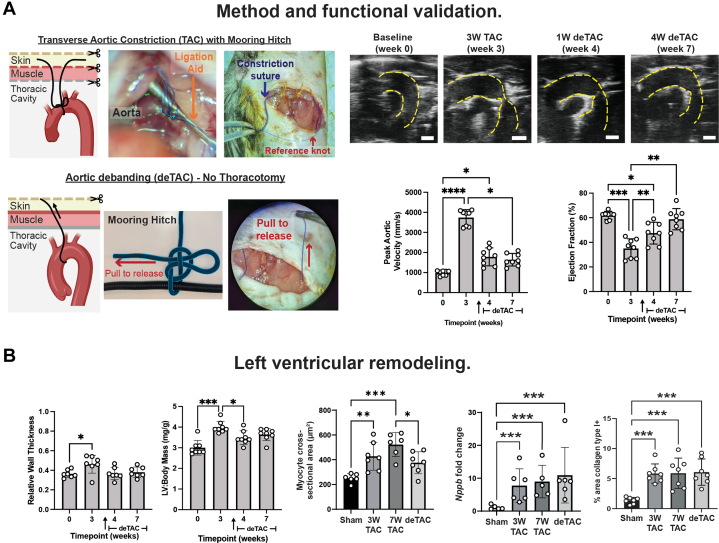
Reversible Transverse Aortic Constriction Methods and Results (A) Schematic and representative images of refined transverse aortic constriction (TAC), minimally invasive de-banding (deTAC), and Mooring hitch and experimental timeline. Black arrows indicate suture traction; scissor icon denotes incised tissue. Representative B-mode images of the transverse aorta (dotted outline) from the same animal at each timepoint. Scale bars represent 1 mm. Peak aortic velocities measured at constriction site by pulsed-wave Doppler. Ejection fraction measured from left ventricular long-axis 2-dimensional B-mode images. (B) Relative wall thickness (2∗[wall thickness/diameter]) measured from 2-dimensional M-mode ultrasound. LV mass measured via 4-dimensional ultrasound. Quantification of myocyte cross-sectional area from WGA staining of the left ventricle. qPCR for Nppb normalized to Gapdh and Actb (2^-ΔΔCt^). Quantification of Collagen type I+ area over total area in the left ventricle. Statistical analyses were performed in GraphPad Prism 10 with significance set at *P <* 0.05. Normality was assessed using the Shapiro-Wilk test. Ejection fraction, left ventricular mass was analyzed using repeated measures analysis of variance with Tukey’s post hoc test. Aortic velocity, relative wall thickness, and normalized left ventricular mass were analyzed using the Friedman test with Dunn’s multiple comparisons. Histology and qPCR data were analyzed by analysis of variance with Tukey’s post hoc text. All values are reported as mean ± SD. ∗*P <* 0.05, ∗∗*P <* 0.01, ∗∗∗*P <* 0.001. Additional details on materials and methods are available upon request.

Eight-week-old male C57Bl/6 mice (22.0 ± 0.9 g, n = 10) were anesthetized, endotracheally intubated, and a left thoracotomy was performed in the second intercostal space to expose the transverse aorta (Figure 1A). All animal experiments followed the University of Montpellier Institutional Animal Care and Use Committee guidelines (protocol number 2023013113485359), European Directive 2010/63/EU on the protection of animals used for scientific purposes, and the Guide for the Care and Use of Laboratory Animals. A 6-0 PVDF suture was positioned beneath the aorta between the brachiocephalic and left common carotid arteries. A 27-gauge blunt needle was placed parallel to the aorta as a spacer, and a mooring hitch knot was tied around both the aorta and the needle. The needle was immediately removed, leaving the constriction in place. An optional square knot was added to end of the release-suture for identification. The suture ends were left extending through the muscle layer, and the thorax and skin were closed with 5-0 resorbable suture (Figure 1A). For deTAC, the mooring hitch knot enabled removal without reopening the thoracic cavity (Figure 1A). Under anesthesia, the skin over the initial incision site was reopened to locate the 2 PVDF suture ends. Gentle traction on one end released the constriction (Figure 1A). The skin was closed with 5-0 resorbable suture. The deTAC procedure took approximately 5 minutes. Following TAC surgery, high-frequency ultrasound (Figure 1A) confirmed successful aortic constriction via elevated blood velocity and visible luminal narrowing (Figure 1A). DeTAC was performed when ejection fraction (EF) was <45%, approximately 3 weeks post-TAC. Ultrasound was performed immediately to verify successful debanding, indicated by re-expansion of the aortic lumen and reduced blood velocity (Figure 1A).

Compared with a traditional definitive knot, the use of a mooring-hitch knot resulted in equivalent remodeling of the ligation site and aortic flow rates (deTAC knot: 3,502 ± 405 mm/s, traditional knot: 4,017 ± 286 mm/s; *P =* 0.091) that were both significantly higher than in sham-operated animals (731 ± 172 mm/s; *P* < 0.001) (as assessed 2 months postsurgery in a separate cohort; n = 4 each group). Blood velocity at the constriction site remained markedly elevated through 3 weeks TAC, and normalized following deTAC. EF declined significantly compared with baseline (62.0 ± 3.2%) at 3 weeks TAC (35.0 ± 7.9%; *P* < 0.001) and progressively recovered after debanding (1 week: 47.5 ± 8.8%; *P =* 0.003; 4 weeks: 58.8 ± 8.6%; *P =* 0.002) (Figure 1A). Relative wall thickness increased compared with baseline (0.36 ± 0.04) at 3 weeks TAC (0.46 ± 0.09; *P =* 0.043) and declined after debanding, although the change was not statistically significant. LV relative mass significantly increased at 3 weeks TAC and partially decreased after deTAC (Figure 1B). In line with this, compared with sham-operated mice, LV myocyte cross-sectional area was significantly increased in mice subjected to 3 or 7 weeks of TAC, but not in the deTAC group (Figure 1B). At the transcript level, hypertrophy marker *Nppb* was up-regulated in LV of TAC and deTAC groups compared with baseline (Figure 1B). Last, compared with sham, we observed a significant increase in the percentage of collagen type I+ area in TAC and deTAC groups (Figure 1B).

In this study, we demonstrate the feasibility of a refined aortic debanding method, performed within 3 weeks after TAC, that enables the investigation of recovery from heart failure (EF <45%) without the need to reopen the chest cavity. Despite functional recovery following debanding, hypertrophy, fibrosis, and elevated expression of hypertrophy markers were observed, suggesting ongoing remodeling in the myocardium as previously reported in the context of reverse remodeling.^3^^,^^4^ Overall, by limiting open surgical intervention, our approach provides a rapid, low-risk, and accessible adaptation of the well-established TAC model.
